# Giant negative magnetoresistance in Manganese-substituted Zinc Oxide

**DOI:** 10.1038/srep09221

**Published:** 2015-03-18

**Authors:** X. L. Wang, Q. Shao, A. Zhuravlyova, M. He, Y. Yi, R. Lortz, J. N. Wang, A. Ruotolo

**Affiliations:** 1 Department of Physics and Materials Science, Device Physics Group, City University of Hong Kong, Kowloon, Hong Kong SAR, China; 2 State Key Laboratory of Superlattices and Microstructures, Institute of Semiconductors, Chinese Academy of Sciences, Beijing 100083, China; 3 Shenzhen Research Institute, City University of Hong Kong, High-Tech Zone, Nanshan District, Shenzhen 518057, China; 4 Department of Physics, The Hong Kong University of Science and Technology, Clear Water Bay, Hong Kong SAR, China

## Abstract

We report a large negative magnetoresistance in Manganese-substituted Zinc Oxide thin films. This anomalous effect was found to appear in oxygen-deficient films and to increase with the concentration of Manganese. By combining magnetoresistive measurements with magneto-photoluminescence, we demonstrate that the effect can be explained as the result of a magnetically induced transition from hopping to band conduction where the activation energy is caused by the *sp-d* exchange interaction.

The *sp - d(f)* exchange interaction between conduction carriers and localized spins in diluted magnetic semiconductors (DMS) gives rise to anomalous optical, magnetic and transport properties. This interaction is at the origin of the giant Faraday and Zeeman effects, first observed in (Cd,Mn)Te^1^,^2^,^3^ and, later, in similar II–VI DMS^4^. Analogous magneto-optical effects were observed in wide band-gap magnetic semiconductors like diluted GaN and ZnO^5^,^6^,^7^. Although much weaker, these effects are still called *giant* because they share the same origin with those measured in II–VI DMS. Remarkably, the effect of the magnetic order on the optical properties of ZnO has been recently demonstrated at room temperature in highly conductive Mn-substituted ZnO films^8^.

Exchange coupling between itinerant carriers and localized spins of magnetic ions is at origin of the anomalous magnetism observed in *n*-type, lightly doped 3*d* diluted magnetic oxide semiconductors, such as ZnO. Ferromagnetic exchange between neighboring magnetic ions can be mediated by the carrier spin if the carrier can delocalize on the magnetic ions with the consequent formation of magnetic polarons^9^. This is possible if an impurity band exists that overlaps (hybridization) with unoccupied *d*-levels of the magnetic dopant. In oxides, this impurity band can be engineered by introducing a large concentration of native oxygen vacancies, which are double donors^10^.

The ability of the conduction electrons to mediate ferromagnetic coupling between localized 4*f* spins gives rise to anomalous change of conductivity in lightly Gd-doped Eu-Chalcogenides^11^. These phenomena have been explained based on a model of hopping conduction of the carriers trapped between magnetic impurity sites with an activation energy strongly dependent on the *s-f* exchange energy^12^. As the temperature is lowered through the Curie temperature or a magnetic field is applied, the conduction mechanism changes from hopping-type to metallic-type impurity band, with a consequent sharp increase of conductivity.

A similar effect should in principle exist, although weaker, in 3*d* transition-metal semiconductors. Indeed, an anomalous negative magnetoresistance has been reported at milli-Kelvin temperatures in (Cd,Mn)Se^13^, (Cd,Mn)Te^14^, (Zn,Mn)Te^15^ and, more surprisingly, in simple Mn-substituted ZnO^16^. Several attempts have been unsuccessfully made to model the effect by using standard theory of spin-disorder scattering, possibly from bound magnetic polarons. But the negative magnetoresistance is always observed for applied magnetic fields well above the saturation magnetization. Moreover, the authors assume that all the donors are activated in the conduction band no matter the temperature, therefore excluding additional formation or annihilation of bound magnetic polarons. Surprisingly the possibility of the existence of an hopping conduction channel is not considered, although hopping conduction at low temperatures has been demonstrated in both ZnO and Mn:ZnO^17^,^18^.

## Results

We here report our study on the negative magnetoresistance in Mn:ZnO thin films. As the temperature is reduced, the resistivity of the films increases with distinct signatures of a transition from band- to hopping-conduction. A sharp decrease of resistance was measured when an external magnetic field was applied. The change of resistivity was found to increase with the concentration of Mn. By using magneto-photoluminescence measurements, we demonstrate that the external magnetic field reactivates the carriers in the conduction band, with a consequent sharp reduction of the film resistivity. In analogy with the case of 4*f* semiconductors, we call the effect *giant* negative magnetoresistance.

The study was carried out on 300 nm thick films of Zn_1−*x*_Mn*_x_*O with *x* = 0 (pure ZnO), 0.02, 0.04 and 0.08 grown by pulsed laser deposition (PLD) on sapphire substrates. Details on the preparation of the targets and films are given in Methods. The films show *n*-type conductivity, regardless temperature. The concentration of oxygen vacancies (V*_O_*'s) in the films was tuned by changing the temperature of the substrate and the oxygen partial pressure during growth. It is relevant here to note that in films with *x* = 0.08 we have carefully excluded the presence of Mn in chemical valence different from 2+ by resorting to low temperature x-ray absorption spectroscopy^19^. Therefore we can reliably exclude that magnetism is due to double-exchange interaction in our films. In addition, photoluminescence (PL) measurements have excluded the presence of Zn vacancies, which are sources of magnetic moment^20^. The films showed a coercivity of ~20 mT and a saturation field of ~200 mT at low temperatures. The large change of magnetoresistance we discuss in the following occurs in a range of fields that extends well above the saturation field.

**Figure 1:**
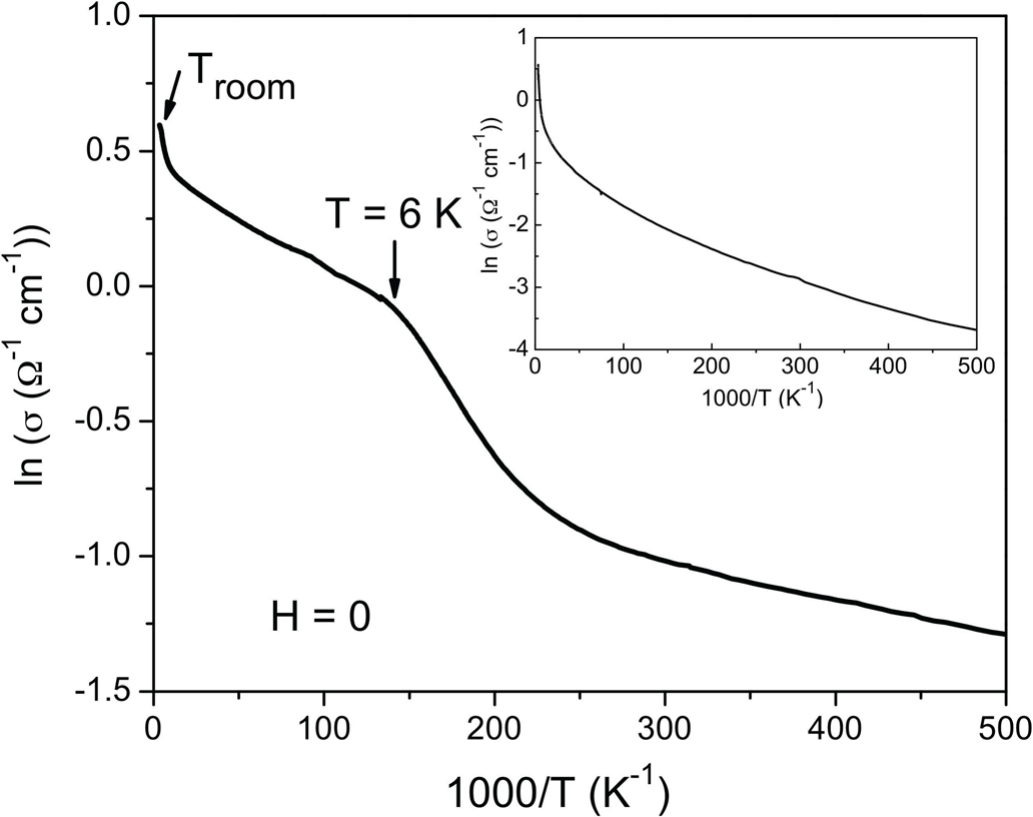
Temperature dependence of the d.c. conductivity of a Mn:ZnO film and a ZnO film (upper-right inset).

Fig. 1 shows the typical temperature dependence of the d.c conductivity of a Mn:ZnO film as compared to that of pure ZnO (shown in the upper-right inset) grown under the same conditions (similar concentration of oxygen vacancies). Mn is isovalent to Zn in ZnO and does not provide additional carriers, therefore the conductivity is supposed to be similar. While the films show similar conductivity at room temperature, ZnO becomes much more resistive at low temperatures. Moreover, two different regimes can clearly be observed in the case of Mn:ZnO. The abrupt change at around T = 6 K indicates a transition from band to hopping conduction.

**Figure 2:**
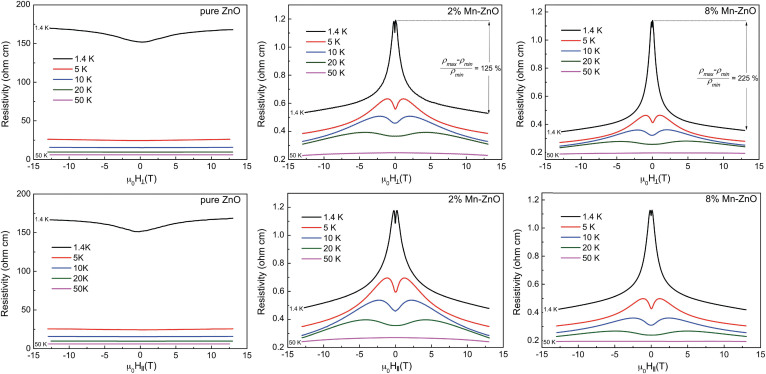
Resistivity as a function of temperature and magnetic field for Zn_1−*x*_Mn*_x_*O with *x* = 0 (pure ZnO), 0.02 and 0.08. The top and bottom rows show the measurement recorded with field applied perpendicular and parallel to the film plane, respectively.

In Fig. 2 we show the resistivity as a function of the applied magnetic field as measured by van der Pauw method for three films of Zn_1−*x*_Mn*_x_*O with, respectively, *x* = 0 (pure ZnO), 0.02 and 0.08, grown under the same conditions. For each sample, we measured the resistivity in magnetic field applied perpendicular to the film plane and parallel to the film plane, as well as current direction. In agreement with Fig. 1, when the temperature is reduced, pure ZnO becomes much more resistive than Mn:ZnO. More interesting, when a magnetic field is applied, the resistivity increases monotonically in pure ZnO while in Mn:ZnO one can distinguish three regimes that partially coexist: the resistance increases at low fields, this increase is overcome by a large decrease of resistance at intermediate fields and, finally, a linear negative magnetoresistance prevails at very high fields.

The positive magnetoresistance observed at low fields depends exponentially on the applied field. This is the characteristic feature of Shklovskii's gigantic magnetoresistance that appears in semiconductors in hopping regime^21^,^22^. The effect is well known and, therefore, will not be discussed in details here. Briefly, the magnetic field squeezes the wave-function of impurity electrons, hence decreasing the overlap of the wave-function tails with a consequent increase of the resistivity. In relatively thick films, which is the case here, the effect is weakly dependent on the relative direction of the current with respect to the field because hopping is a percolative process. The appearance of Shklovskii's magnetoresistance is relevant here because it proves that, at low temperatures, hopping conduction becomes the dominant conduction mechanism in our films. Moreover, a much lower resistivity in hopping regime of the Mn:ZnO as compared to pure ZnO suggests that in Mn:ZnO hopping occurs between Mn sites, whereas it occurs between crystal defects in pure ZnO^17^.

As the magnetic field increases, a dramatic reduction of the resistivity is observed in Mn:ZnO that overcomes the Shklovskii's positive magnetoresistance. More precisely, the field destroys the dramatic increase of resistivity, an increase that was due to the freezing out of carriers when cooling down. It is therefore straightforward to conclude that the applied field is able to drive the system from hopping- to band- conduction. Such a strong, anisotropic, dependence of the activation energy on the applied magnetic field suggests a physical mechanism similar to that behind the giant negative magnetoresistance observed in doped Eu-chalcogenides. In the following we briefly recall the microscopic scenario for the case of Eu_1−*x*_Gd*_x_*Se and, then, we discuss the fundamental differences between these alloys and Mn:ZnO.

A large decrease of resistivity is observed when Eu_1−*x*_Gd*_x_*Se is cooled through its Curie temperature^11^. This anomalous phenomenon has been well studied and explained^12^. Gd substitutes Eu^2+^ with valence 3+ and the excess electrons form an impurity band that overlaps with the conduction band of EuSe. At room temperature the impurity electrons are thermally excited in the conduction band. As the temperature is reduced, the electrons localize on the Gd ions and a hopping conduction channel appears. In an occupied site, the impurity *s* electron will mediate exchange interaction between the 4*f* electrons of the Gd^3+^, as well as the 4f electrons of the surrounding Eu^2+^. As the impurity electron gains the energy of the *s-f* exchange interaction, its activation energy increases. Hopping requires large activation energy because the 4*f* spins in the unoccupied sites are randomly oriented. Yet, if magnetic order appears, or an external magnetic field is applied that aligns the 4*f* spins, the activation energy sharply decreases and so does the resistivity.

Mn is isovalent to Zn in the wurtzite ZnO crystal and, unlike Gd in EuSe, is not a dopant. The carrier spins that mediate exchange interaction are provided by the V*_O_*'s. Exchange interaction is established when a *p* electron from a V*_O_* delocalizes on a Mn^2+^ energy level and mediates exchange interaction between *d* electrons of the Mn^2+^ sites^9^. More properly one should think of this process as a delocalization on a Mn-V*_O_* complex, rather than on a Mn ion. The chemical valence of the Mn will remain 2+. The magnetic alignment is not due to double-exchange interaction between Mn in mixed valence but formation of Mn-V*_O_*-Mn polarons. As the temperature is reduced, impurity electrons from V*_O_*'s start freezing out and a hopping channel is established. Hopping occurs between Mn-V*_O_* complexes that are randomly oriented, therefore belonging to different polarons. A magnetic field that tends to align Mn-V*_O_* complexes results in a sharp reduction of the resistivity. Unlike the case of Eu_1−*x*_Gd*_x_*Se, the effect is not limited to the temperature range near the Curie temperature because of the fundamentally different mechanism of magnetic interaction in the two materials. Mn:ZnO does not have a sharp magnetic transition, if a magnetic transition can be defined at all. The molecular field plays a marginal role as compared to the externally applied field in the case of Mn:ZnO and the giant negative magnetoresistance exists as long as an hopping channel can be created. The peculiar form of magnetism in Mn:ZnO that has confined this material to mere academic curiosity might represent its uniqueness and fortune when it comes to magneto transport properties, with potential applications in magnetic sensing.

**Figure 3:**
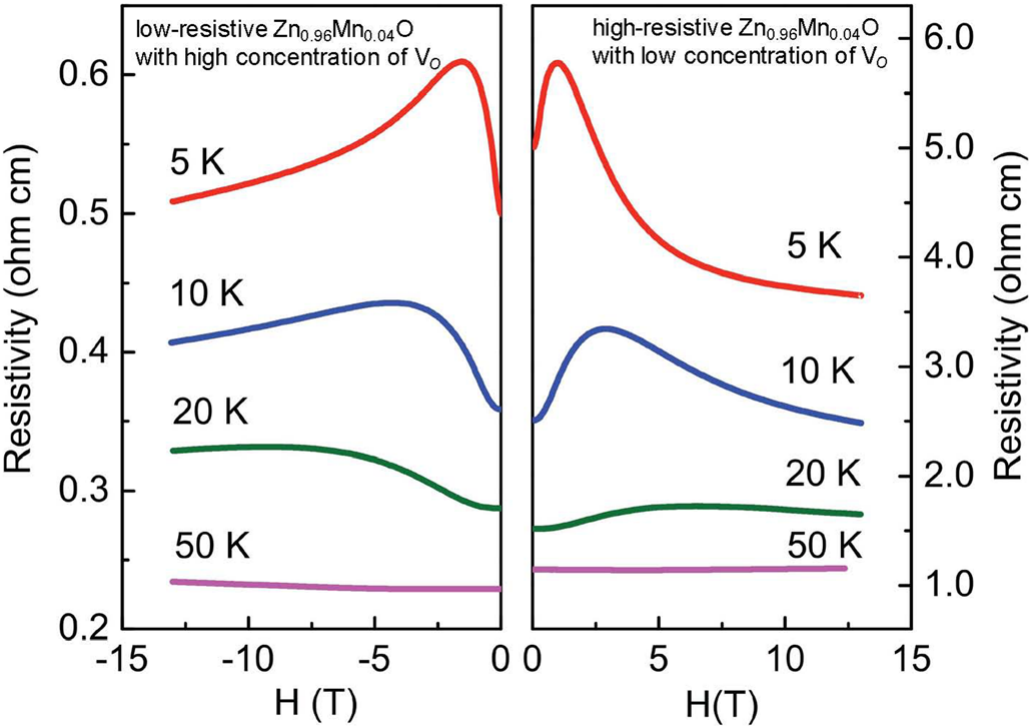
Resistivity as a function of temperature and magnetic field for Zn_1−*x*_Mn*_x_*O with *x* = 0.04 and different concentrations of V_O_'s.

In this respect a great challenge will be to tune the concentration of V*_O_*'s in the material to maximize the negative magnetoresistance. A small concentration of V*_O_*'s would make hopping energetically unfavorable, whereas an excessive number of V*_O_*'s will result in a band-conduction channel that shorts the hopping-channel. In Fig. 3 we compared the magnetoresistive behavior of two films of Zn_1−*x*_Mn*_x_*O with *x* = 0.04, grown under two different oxygen pressures. A lower oxygen pressure during growth corresponds to a larger concentration of V*_O_*'s and therefore a smaller resistivity. As the temperature is reduced, the density of activated electrons in the conduction band is larger for the case of low resistive Mn:ZnO. As a consequence, its resistivity remains lower and so does the giant negative magnetoresistance.

**Figure 4:**
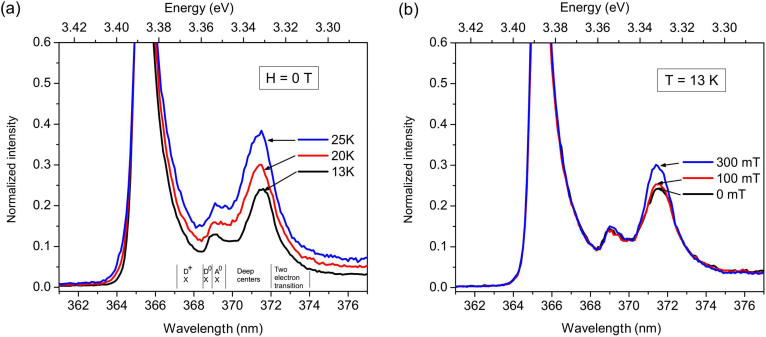
Normalized PL spectra of a Zn_0.92_Mn_0.08_O film at (a) increasing temperatures and (b) increasing magnetic field.

Unlike the case of Eu_1−*x*_Gd*_x_*Se, in the wide-band gap, transparent Mn:ZnO the ability of the external field to activate carriers localized on the Mn-V*_O_* complexes can be probed by resorting to magneto-photoluminescence. In fact, V*_O_*'s are optically active defect centers that can form mono centric or pair exciton complexes at low temperatures. In Fig. 4(a) we show the normalized PL spectra recorded at various temperatures for a film of Zn_1−*x*_Mn*_x_*O with *x* = 0.08. Besides the fundamental near-band-edge peak, we detect a peak centered at λ = 371.5 nm that cannot be attributed to neutral or ionized donors/acceptors. Peaks in this region can be attributed to two-electron transitions^23^ or excitons being bound to defect pairs^24^, where the term defect includes intentional or native dopants. The broadness of the peak, which is comparable to that of the main PL peak, seems to exclude a two-electron transition, which show sharp peaks in ZnO. In the case of pair transitions, the PL peaks are relatively broad because the energy of the bound exciton depends on the donor-acceptor pair, which is unlikely to be the same for all the pairs. In insulators and wide-band gap semiconductors, vacancies can lead to the formation of deep-level *F*-centres^25^,^26^. These centers consist of electrons trapped at the place of a missing charged ion and can be treated theoretically as an electron trapped in a finite dipole^27^. Exchange interaction between electrons trapped at nearby vacancies has also been theoretically considered for the case of paramagnetic MgO and CaO^28^. The case of *F*-centers in magnetic polarons can be far more complex.

## Discussion

An electron localized on an Mn-V*_O_* pair can appear as a deep *F*-center in the low-temperature PL spectrum of oxygen-deficient Zn_1−*x*_Mn*_x_*O. *F*-centers follow the Mollwo-Ivey relation^25^,^29^:
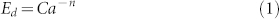
where *E_d_* is the absorption energy and *a* is the average inter atomic separation of the pair in Angstrom. This relation was empirically derived for alkali halides, for which values of the parameters *n* = 1.81 and *C* = 17.3 ± 2.8 eV well fitted the experimental data^30^. In the case of oxides, such as MgO, CaO and SrO, the equation works well if a factor *n* = 2.4 is used^26^. Assuming a separation *a* = 2.0 Å, which corresponds to the Zn-O bond length and *E_d_* = 3.33 eV, eq. 1 yields *C* = 17.6 eV. Let us also notice that luminescence bands for *F*-centers in MgO and CaO have been reported at 3.31 eV and 3.32 eV, respectively^26^. While the effect of a magnetic field on the bound exciton complexes in pure ZnO has been previously studied^31^,^32^, the problem of the excitation of Mn-V*_O_* complexes in ferromagnetic ZnO is far more complicated and certainly beyond the scope of this Report. However, in order to verify the validity of the model previously outlined for the microscopic description of the observed giant negative magnetoresistance, we measured the PL spectrum in magnetic field. As it can be appreciated from Fig. 4, the magnetic field has a similar effect as the temperature on the Mn-V*_O_* complexes. Electrons localized on the complexes can be activated in the conduction band by either increasing the temperature (see Fig. 4(a)) or applying a magnetic field (see Fig. 4(b)). Due to the strong *sp-d* exchange interaction, and therefore *giant* Zeeman splitting, delocalization occurs even for field of the order of hundreds of mT. The activation of these electrons, whether due to the temperature or the magnetic field results in a *giant* reduction of resistivity (see Fig. 2).

Let us finally comment on the weak, linear negative magnetoresistance which becomes the dominant regime at very high magnetic fields (see Fig. 2). This can be simply attributed to magnetic scattering processes on Mn^2+^ ions in paramagnetic or antiferromagnetic state. It has been well established that not all the magnetic ions form polarons^9^. Part of the dopant is in paramagnetic state or form antiferromagnetic pairs through mediation by oxygen. In our films, the typical saturation magnetic moment per Mn atom in Mn:ZnO is of the order of 1 *μ_B_*/Mn^2+^ in films with high concentration of V*_O_*^10^. This value is smaller than the theoretical value of 5 *μ_B_*/Mn^2+^. This confirms that significant amount of Mn ions are not forming magnetic polarons through the mediation of V*_O_*'s.

## Methods

### Film preparation

The films of Zn_1−*x*_Mn*_x_*O with *x* = 0 (pure ZnO), 0.02, 0.04 and 0.08 were grown by pulsed laser deposition (PLD) on Al_2_O_3_ <0001> crystal substrates from three different targets prepared by solid state reaction method from ZnO and MnO_2_ powders mixed according to the desired stoichiometry. The powders were mixed, grounded by ball milling for 10 h, and sintered at 400°C for 8 h, and then at 600°C for 12 h. A pulsed KrF excimer laser (*k* = 248 nm) was used for the films growth with a repetition rate of 10 Hz and energy 300 mJ. Four sets of films were grown from each target in high vacuum (10^−5^ mbar) at room temperature.

### Characterization

The structure of the films was studied by an x-ray diffractometer (Philps X'Pert) with a Cu Ka radiation source (*k*_Cu_ = 0.15406 nm). The films were highly texturized with a grain size of about 300 nm, as calculated from the Full-Width Half-Maximum of the (002) main peak by using Debye-Scherrer formula. No significant strain could be deduced from the (002) peak position. The stoichiometry of all the targets and the films was checked by energy dispersive spectroscopy. Low temperature x-ray absorption spectroscopy were carried out on the films with *x* = 0.08 to verify absence of Mn in valence different from 2+. The surface morphology was investigated by using an Atomic Force Microscope. The chip with the devices was connected through wire bonding to standard laboratory electronics with applied magnetic field up to 15 T and temperature down to 1.4 K for d.c. transport and magnetoresistance characterization by van der Pauw method. Low temperature photoluminescence measurements under external fields were carried out by putting a small permanent magnet inside the chamber.

## Author Contributions

A.R. coordinated the work and prepared the manuscript. X.L.W. and Q.S. fabricated the samples. X.L.W., A.Z., M.H. and R.L. carried out the transport measurements. Y.Y., S.Q. and J.N.W. carried out the photoluminescence measurements in magnetic field. X.L.W., Q.S. and A.R. analyzed the data. All authors discussed the results. X.L.W. and Q.S. contributed equally to this paper.

## References

[b1] Komarov A. V., Ryabchenko S. M., Terletskii O. V., Zheru I. I. & Ivanchuk R. D. Magneto-optical investigations of the exciton band in CdTe:Mn^2+^. Zh. Eksp. Teor. Fiz. 73, 608–618 (1977).

[b2] Gaj J. A., Galazka R. R. & Nawrocki M. Giant exciton faraday rotation in Cd_1−x_Mn_x_Te mixed crystals. Solid State Commun. 25, 193 (1978).

[b3] Twardowski A., Nawrocki M. & Ginter J. Excitonic magnetoabsorption in Cd_1−x_Mn_x_Te mixed crystals. Phys. Stat. Sol. B 96, 497–506 (1979).

[b4] Gaj J. A. & Kossut J. Introduction to the Physics of Diluted Magnetic Semiconductors. Springer Series in Materials Science (Springer, 2010).

[b5] Pacuski W. *et al.* Excitonic giant Zeeman effect in GaN: Mn^3+^. Phys. Rev. B 76, 165304 (2007).

[b6] Pacuski W. *et al.* Observation of Strong-Coupling Effects in a Diluted Magnetic Semiconductor Ga_1−x_Fe_x_N. Phys. Rev. Lett. 100, 037204 (2008). 18233033 10.1103/PhysRevLett.100.037204

[b7] Pacuski W. *et al.* Effect of the *s, p-d* exchange interaction on the excitons in Zn_1−x_Co_x_O epilayers. Phys. Rev. B 73, 035214 (2006).

[b8] Wang X. L. *et al.* Effect of the magnetic order on the room-temperature band-gap of Mn-doped ZnO thin films. Appl. Phys. Lett. 102, 102112 (2013).

[b9] Coey J. M. D., Venkatesan M. & Fitzgerald C. B. Donor impurity band exchange in dilute ferromagnetic oxides. Nat. Mater. 4, 173–179 (2005). 15654343 10.1038/nmat1310

[b10] Wang X. L., Lai K. H. & Ruotolo A. A comparative study on the ferromagnetic properties of undoped and Mn-doped ZnO. J. Alloys Compd. 542, 147–150 (2012).

[b11] Von Molnar S. & Methfessel S. Giant Negative Magnetoresistance in Ferromagnetic Eu_1−x_Gd_x_Se. J. Appl. Phys. 38, 959–964 (1967).

[b12] Kasuya T. & Yanase A. Anomalous Transport Phenomena in Eu-Chalcogenide. Alloys. Rev. Mod. Phys. 40, 684–696 (1968).

[b13] Sawicki M. *et al.* Influence of *s-d* Exchange Interaction on the Conductivity of Cd_1−x_Mn_x_Se:In in the Weakly Localized Regime. Phys. Rev. Lett. 56, 508–511 (1986). 10033210 10.1103/PhysRevLett.56.508

[b14] Leighton C., Terry I. & Becla P. Metallic conductivity near the metal-insulator transition in Cd_1−x_Mn_x_Te. Phys. Rev. B 58, 9773–9782 (1998).

[b15] Van Khoi L. *et al.* Growth and Electrical Properties of Phosphorus Doped Zn_1−x_Mn_x_Te Crystals. Acta Phys. Polon. A 92, 833–836 (1997).

[b16] Andrearczyk T. *et al.* Spin- related magnetoresistance of *n*-type ZnO:Al and Zn_1−x_Mn_x_O:Al thin films. Phys. Rev. B 72, 121309 (2005).

[b17] Schoenes J., Kanazawa K. & Kay E. Band and hopping conduction in high-resistivity ZnO. J. Appl. Phys. 48, 2537–2542 (1977).

[b18] Han J., Shen M. & Cao W. Hopping conduction in Mn-doped ZnO. App. Phys. Lett. 82, 67–69 (2003).

[b19] Shao Q. *et al.* Chemical states and ferromagnetism in heavily Mn-substituted zinc oxide thin films. J. Appl. Phys. 115, 153902 (2014).

[b20] Yi J. B. *et al.* Ferromagnetism in Dilute Magnetic Semiconductors through Defect Engineering: Li-Doped ZnO. Phys. Rev. Lett. 104, 137201 (2010). 20481907 10.1103/PhysRevLett.104.137201

[b21] Pollak M. & Shklovskii B. Hopping Transport in Solids (Amsterdam: North-Holland, 1991).

[b22] Shklovskii B. & Efros A. Electronic properties of doped semiconductors (Springer- Verlag, 1984).

[b23] Reynolds D. C. & Collins T. C. Excited Terminal States of a Bound Exciton-Donor Complex in ZnO. Phys. Rev. 185, 1099–1103 (1969).

[b24] Tomzig E. & Helbig R. Band-edge emission in ZnO. J. Lumin. 14, 403–415 (1976).

[b25] Mollwo E. Über die Farbzentren der Alkalihalogenidkristalle. Z. Physik. 85, 56–57 (1933).

[b26] Henderson B. Anion vacancy centers in Alkaline-Earth oxides. Crit. Rev. Solid State Mater. Sci. 9, 1–60 (1980).

[b27] To K. C., Stoneham A. M. & Henderson B. Electron Spin Resonance from a Vacancy-Pair Center in Magnesium Oxide. Phys. Rev. 181, 1237–1240 (1969).

[b28] Norgett M. J. Electron Spin Resonance from a Vacancy-Pair Center in Magnesium Oxide. J. Phys. C: Solid State Phys. 4, 1289–1298 (1971).

[b29] Ivey H. F. Spectral Location of the Absorption Due to Color Centers in Alkali Halide Crystals. Phys. Rev. 72, 341–343 (1947).

[b30] Dawson R. K. & Pooley D. F Band Absorption in Alkali Halides as a Function of Temperature. Phys. Stat. Sol. B 35, 95–105 (1969).

[b31] Rodina A. V. *et al.* Magneto-optical properties of bound excitons in ZnO. Phys. Rev. B 69, 125206 (2004).

[b32] Ding L. *et al.* Classification of bound exciton complexes in bulk ZnO by magnetophotoluminescence spectroscopy. J. Appl. Phys. 105, 053511 (2009).

